# Supplementary figures and images for: New seed coating containing Trichoderma viride with anti-pathogenic properties
Source: PeerJ. 2023 Jun 1;11:e15392. doi: 10.7717/peerj.15392 (PMC10239620; doi:10.7717/peerj.15392)

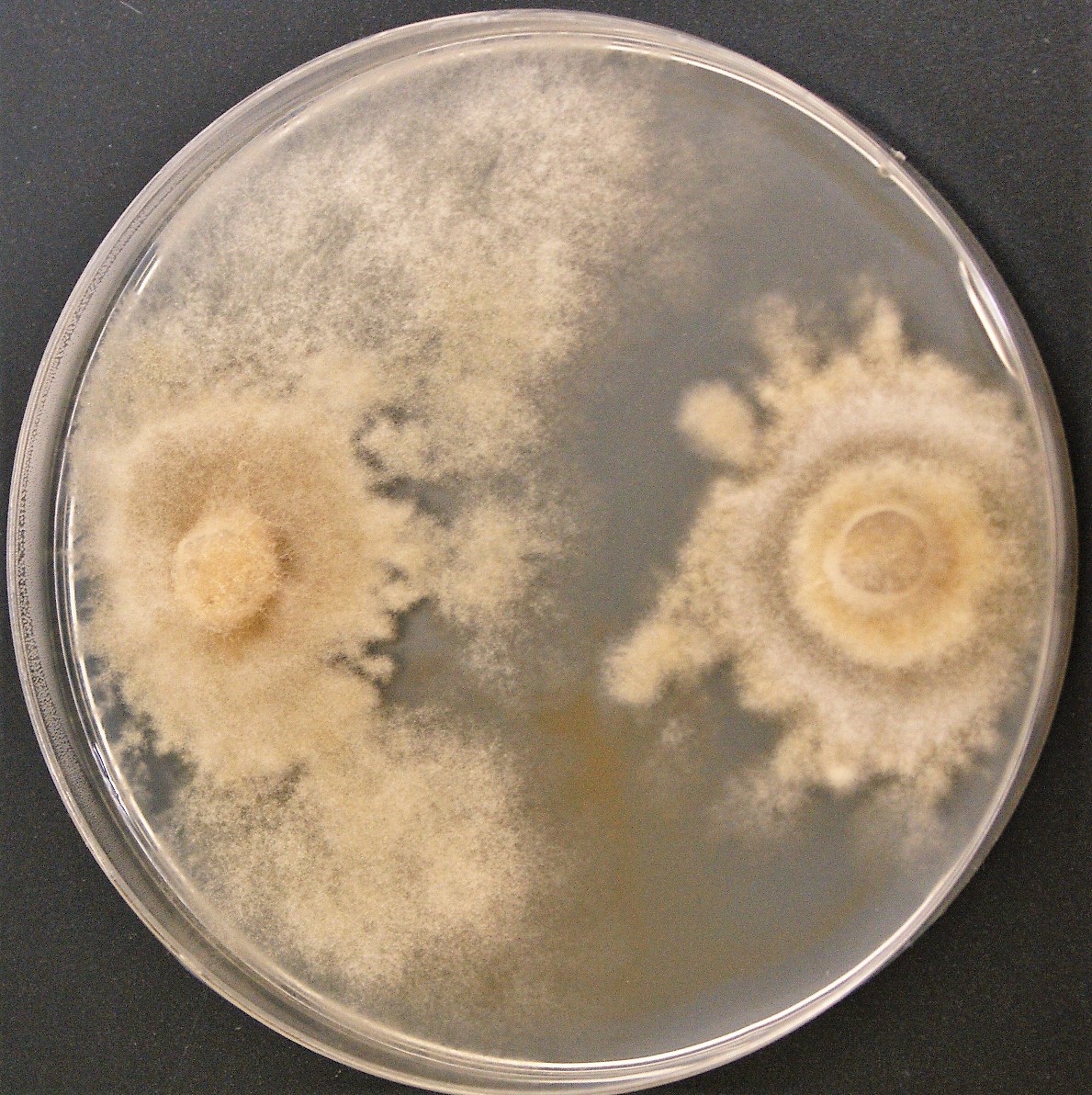

Supplement: Supplemental Information 1 — Images depict comparative growth of multiple fungal variants (labelled), thereby indicating the inhibitory potential of Trichoderma. [file peerj-11-15392-s001.zip › Photos/B. cinerea-B. cinerea.jpg]

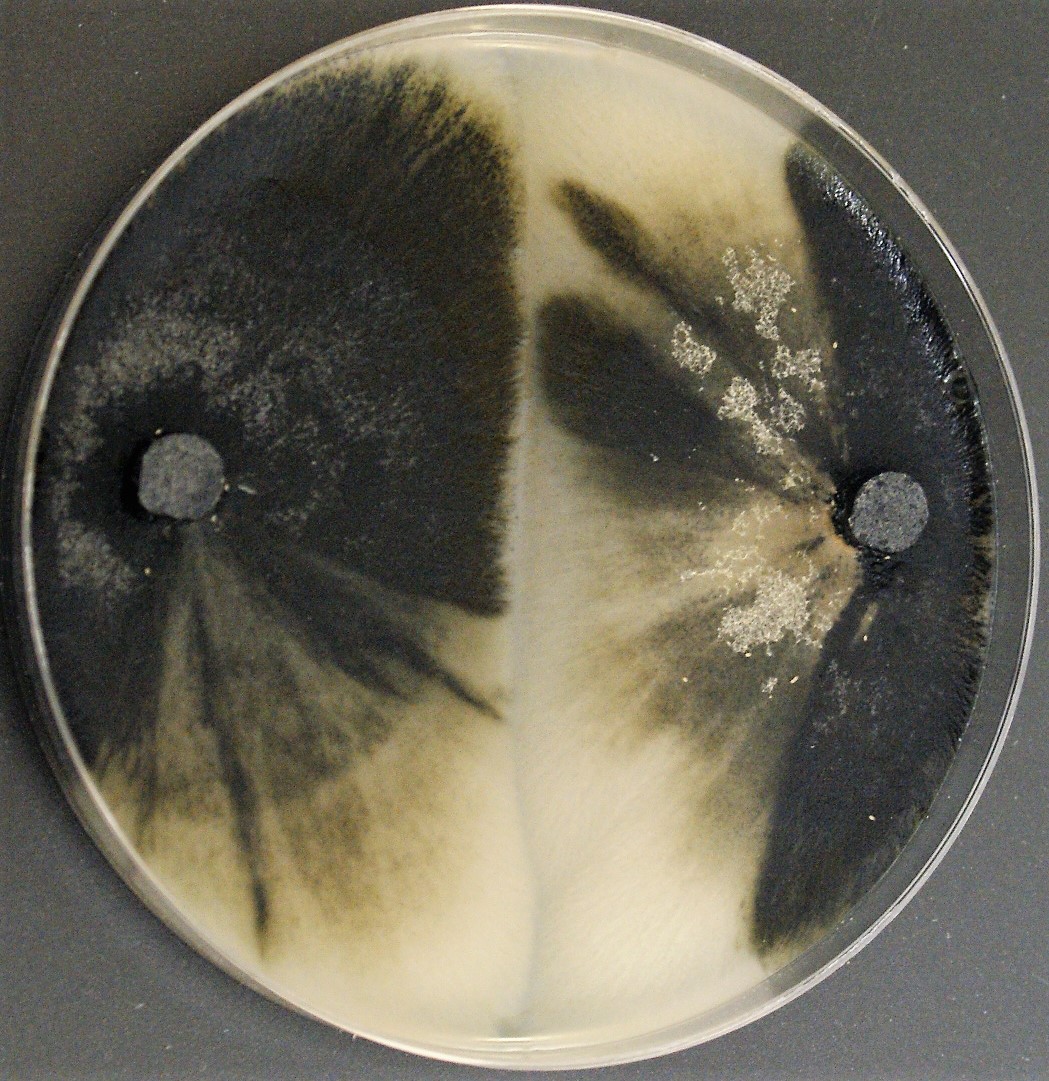

Supplement: Supplemental Information 1 — Images depict comparative growth of multiple fungal variants (labelled), thereby indicating the inhibitory potential of Trichoderma. [file peerj-11-15392-s001.zip › Photos/Colletotrichum-Colletotrichum.jpg]

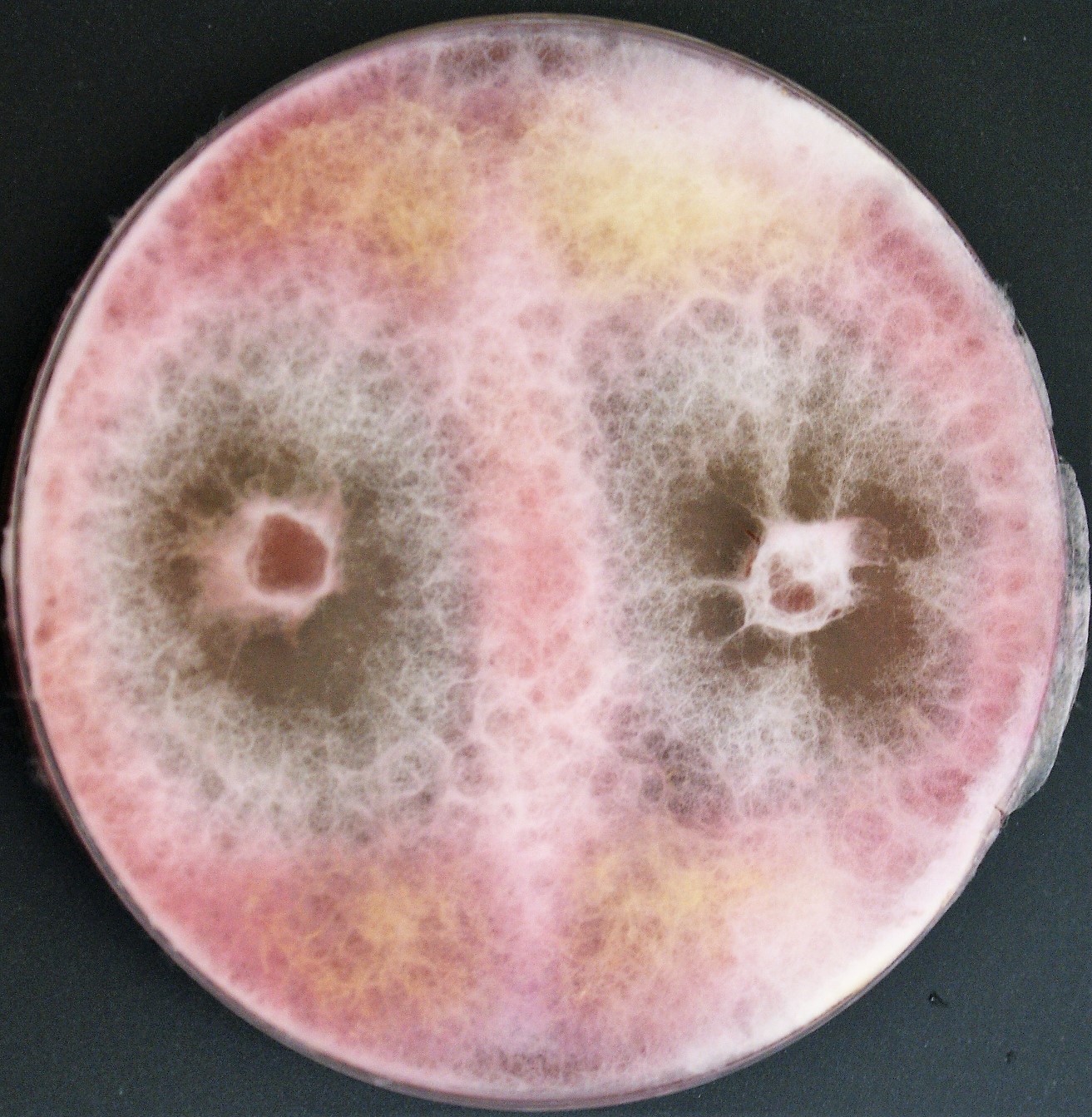

Supplement: Supplemental Information 1 — Images depict comparative growth of multiple fungal variants (labelled), thereby indicating the inhibitory potential of Trichoderma. [file peerj-11-15392-s001.zip › Photos/F. culmorum-F. culmorum.jpg]

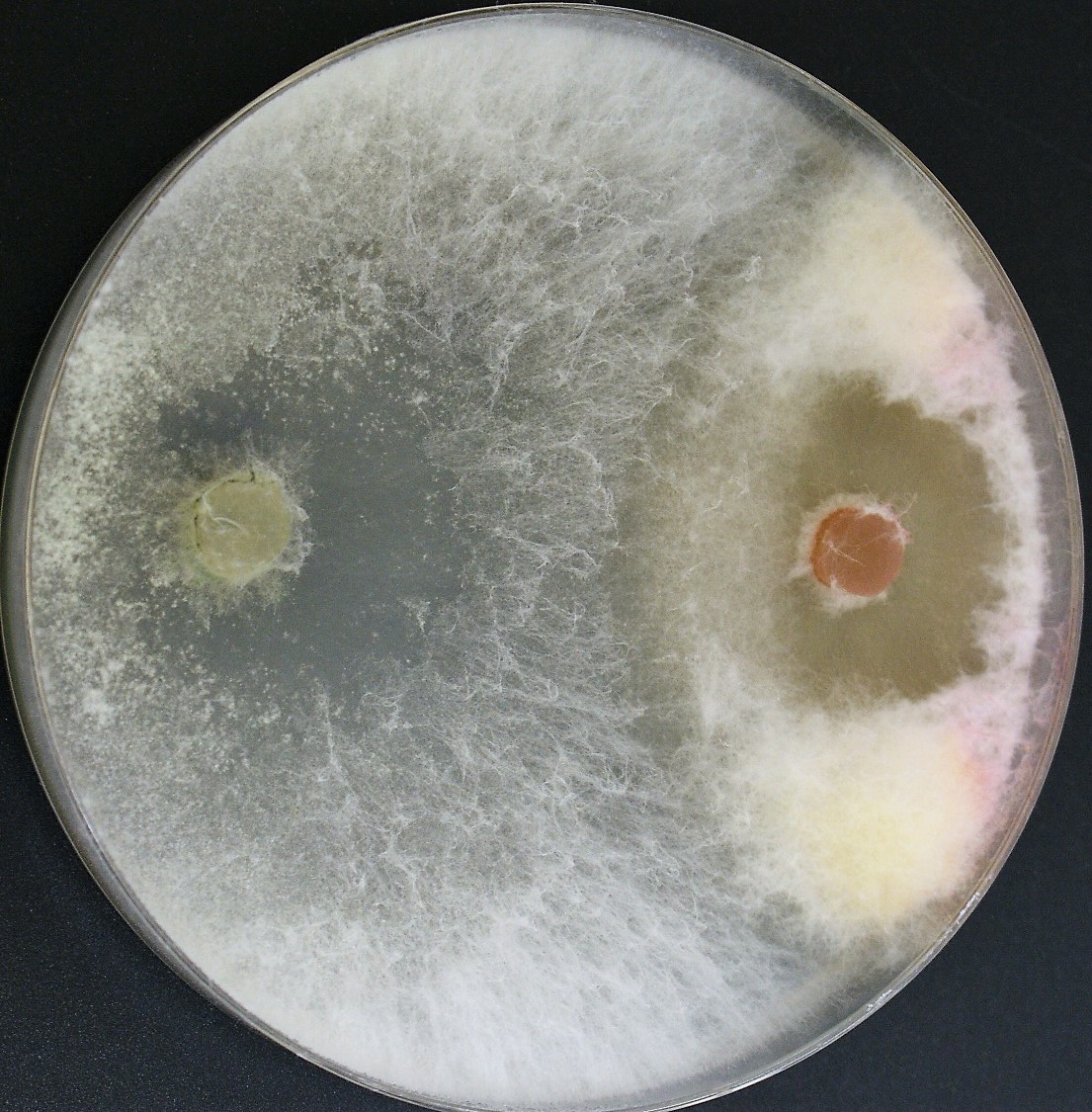

Supplement: Supplemental Information 1 — Images depict comparative growth of multiple fungal variants (labelled), thereby indicating the inhibitory potential of Trichoderma. [file peerj-11-15392-s001.zip › Photos/T. viride I-F. culmorum.jpg]

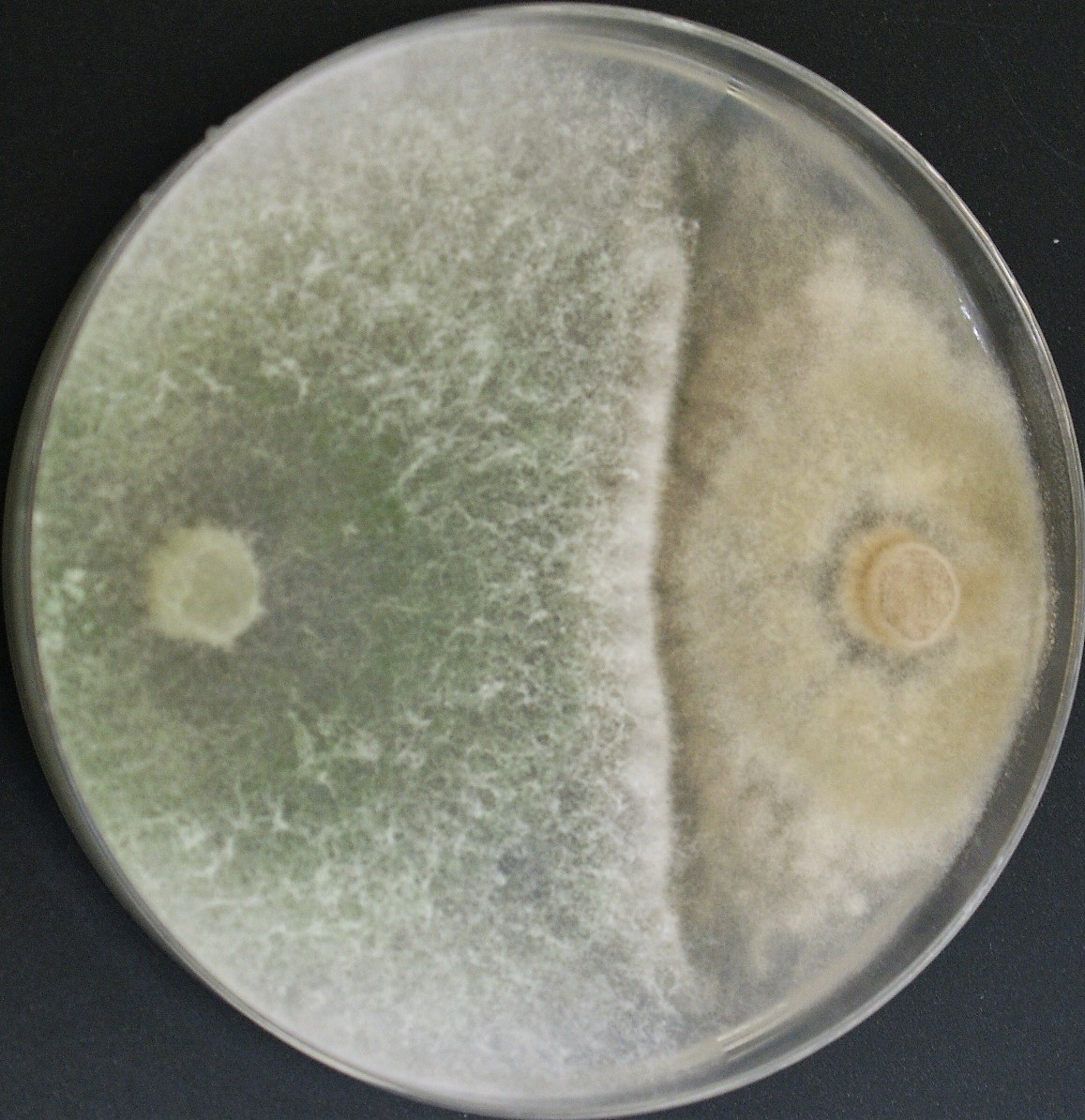

Supplement: Supplemental Information 1 — Images depict comparative growth of multiple fungal variants (labelled), thereby indicating the inhibitory potential of Trichoderma. [file peerj-11-15392-s001.zip › Photos/T. viride II-B.cinerea.jpg]

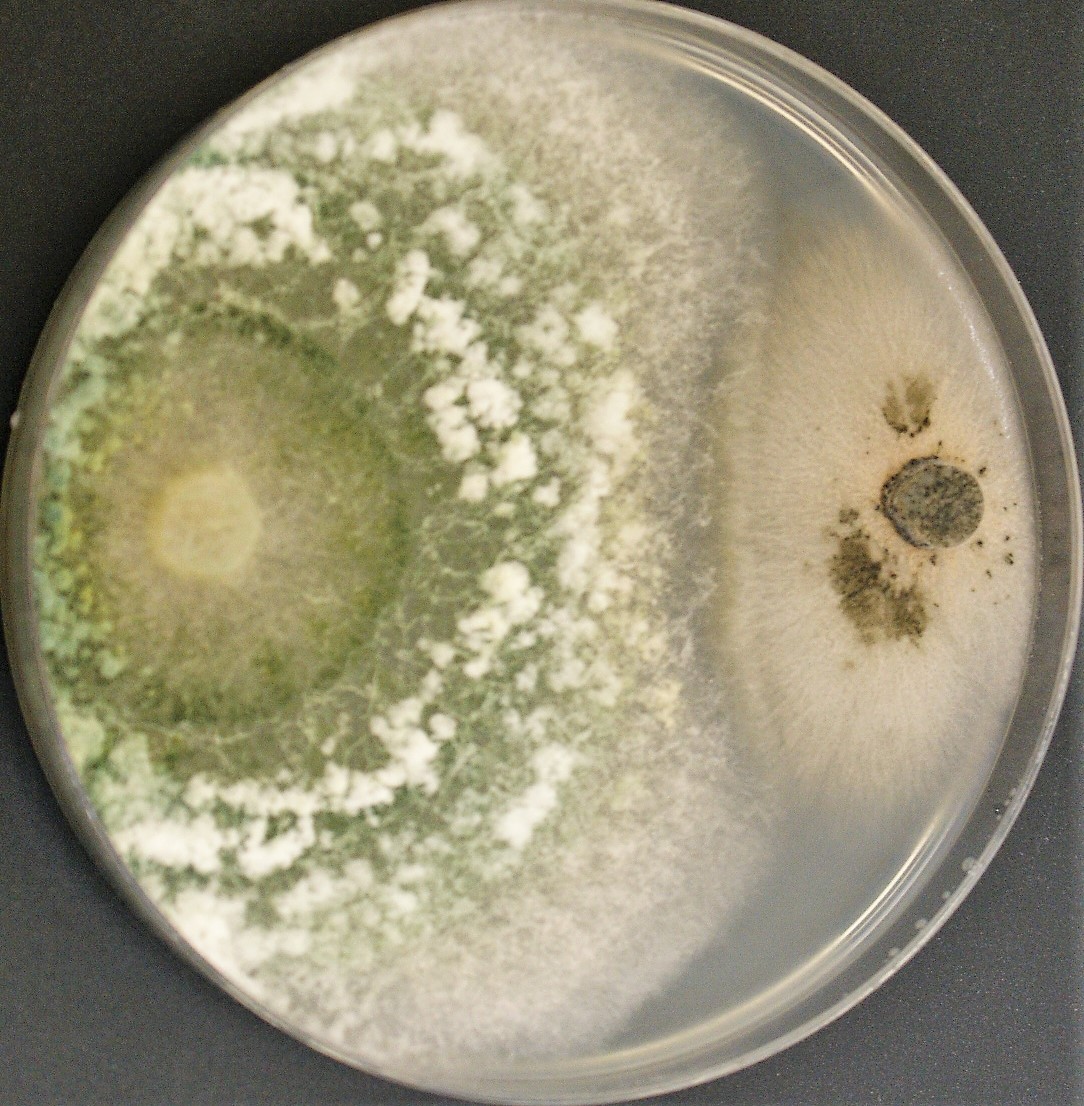

Supplement: Supplemental Information 1 — Images depict comparative growth of multiple fungal variants (labelled), thereby indicating the inhibitory potential of Trichoderma. [file peerj-11-15392-s001.zip › Photos/T. viride II-Colletotrichum.jpg]

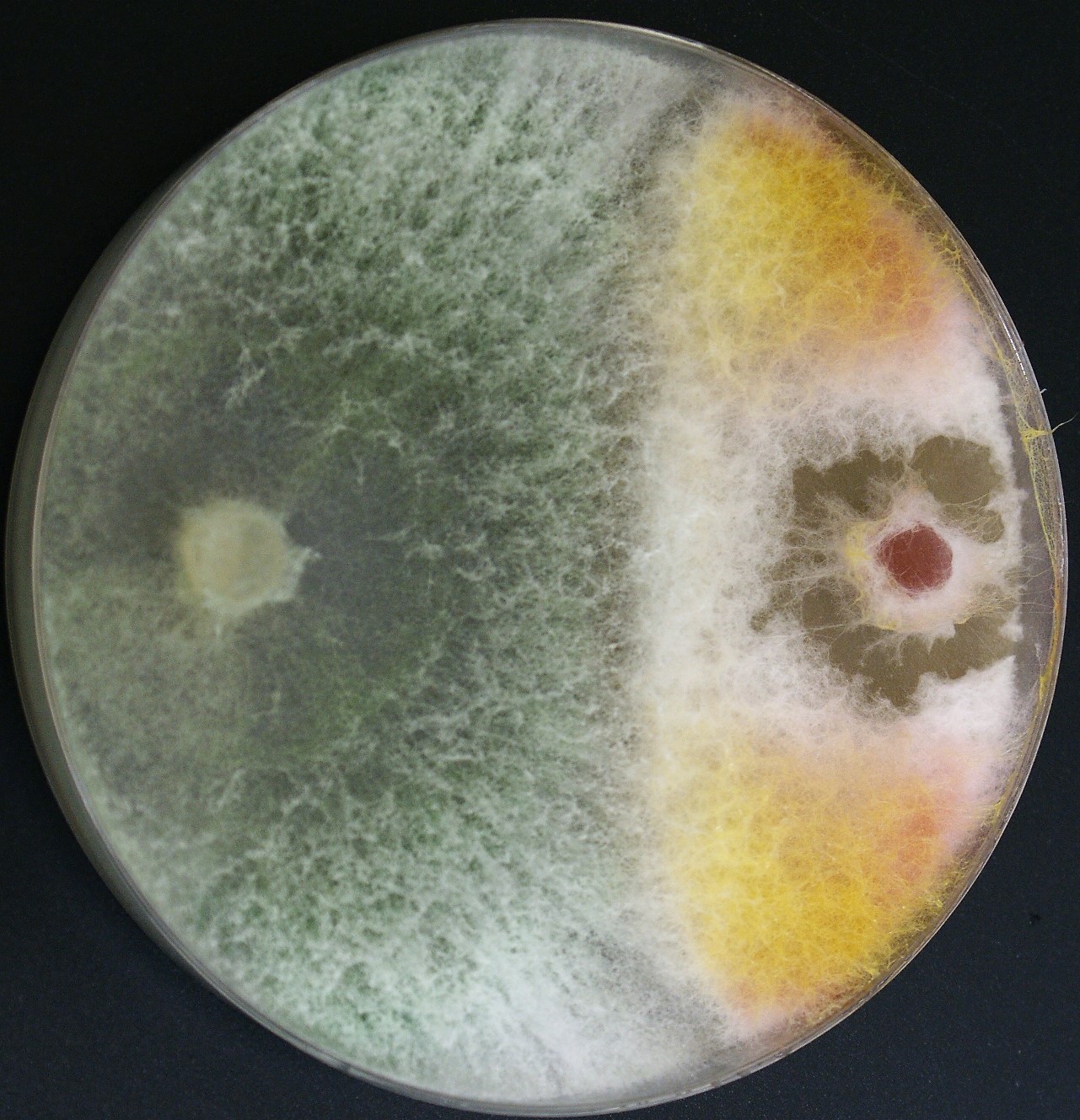

Supplement: Supplemental Information 1 — Images depict comparative growth of multiple fungal variants (labelled), thereby indicating the inhibitory potential of Trichoderma. [file peerj-11-15392-s001.zip › Photos/T. viride II-F. culmorum.jpg]

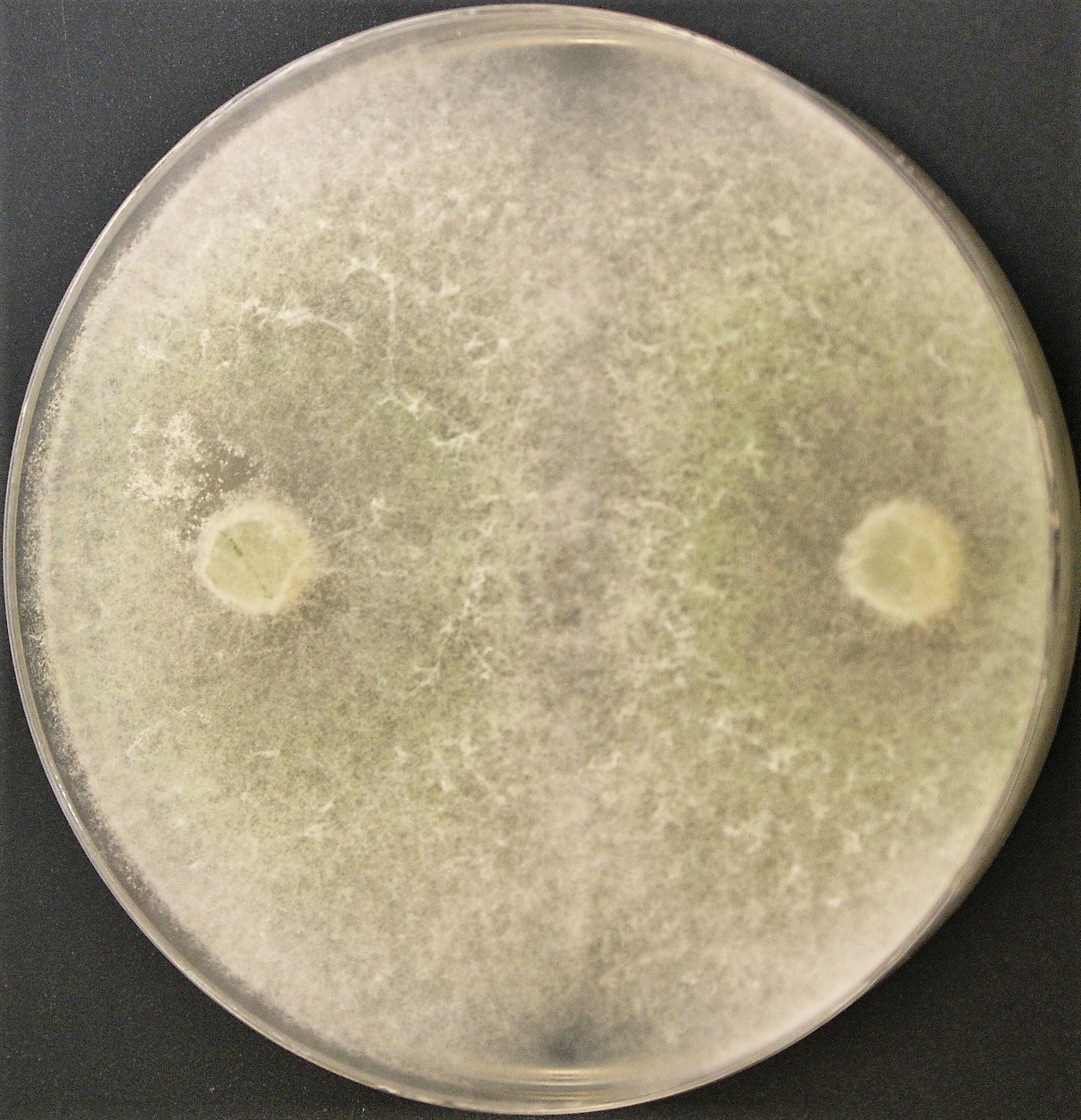

Supplement: Supplemental Information 1 — Images depict comparative growth of multiple fungal variants (labelled), thereby indicating the inhibitory potential of Trichoderma. [file peerj-11-15392-s001.zip › Photos/T. viride II-T. viride II.jpg]

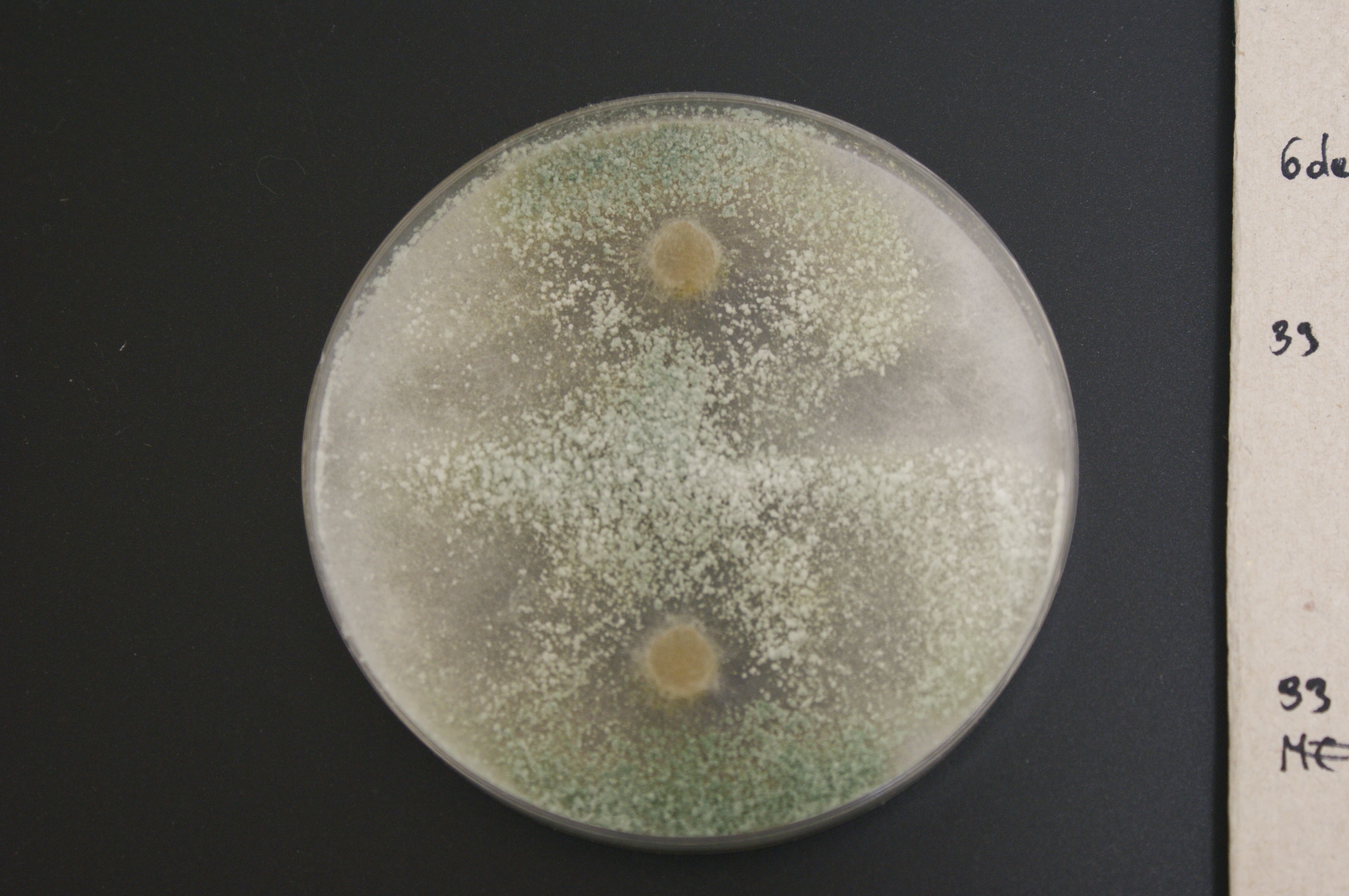

Supplement: Supplemental Information 1 — Images depict comparative growth of multiple fungal variants (labelled), thereby indicating the inhibitory potential of Trichoderma. [file peerj-11-15392-s001.zip › Photos/T. viride I-T. viride I.JPG]

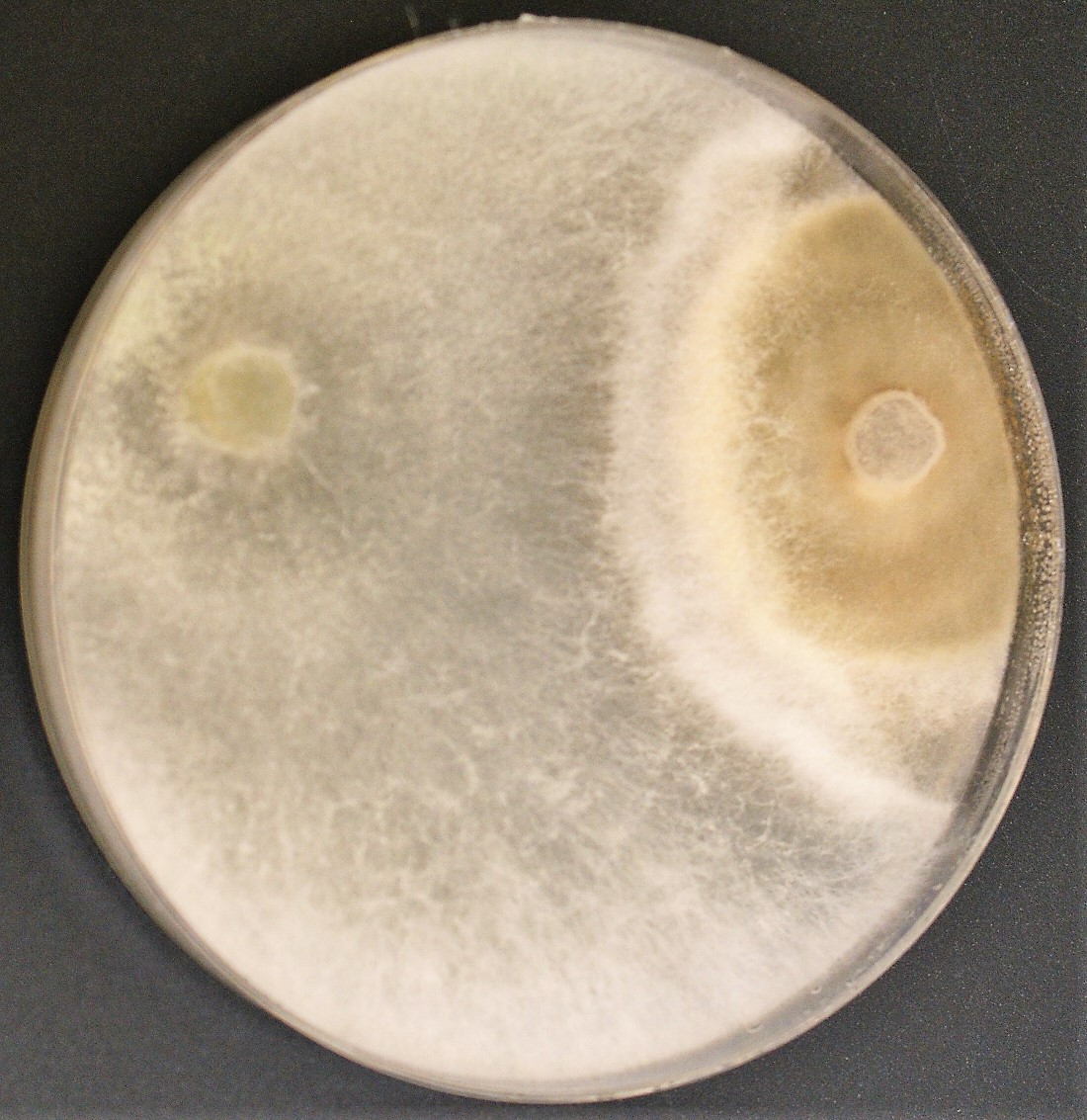

Supplement: Supplemental Information 1 — Images depict comparative growth of multiple fungal variants (labelled), thereby indicating the inhibitory potential of Trichoderma. [file peerj-11-15392-s001.zip › Photos/T.virideI-B. cinerea.jpg]

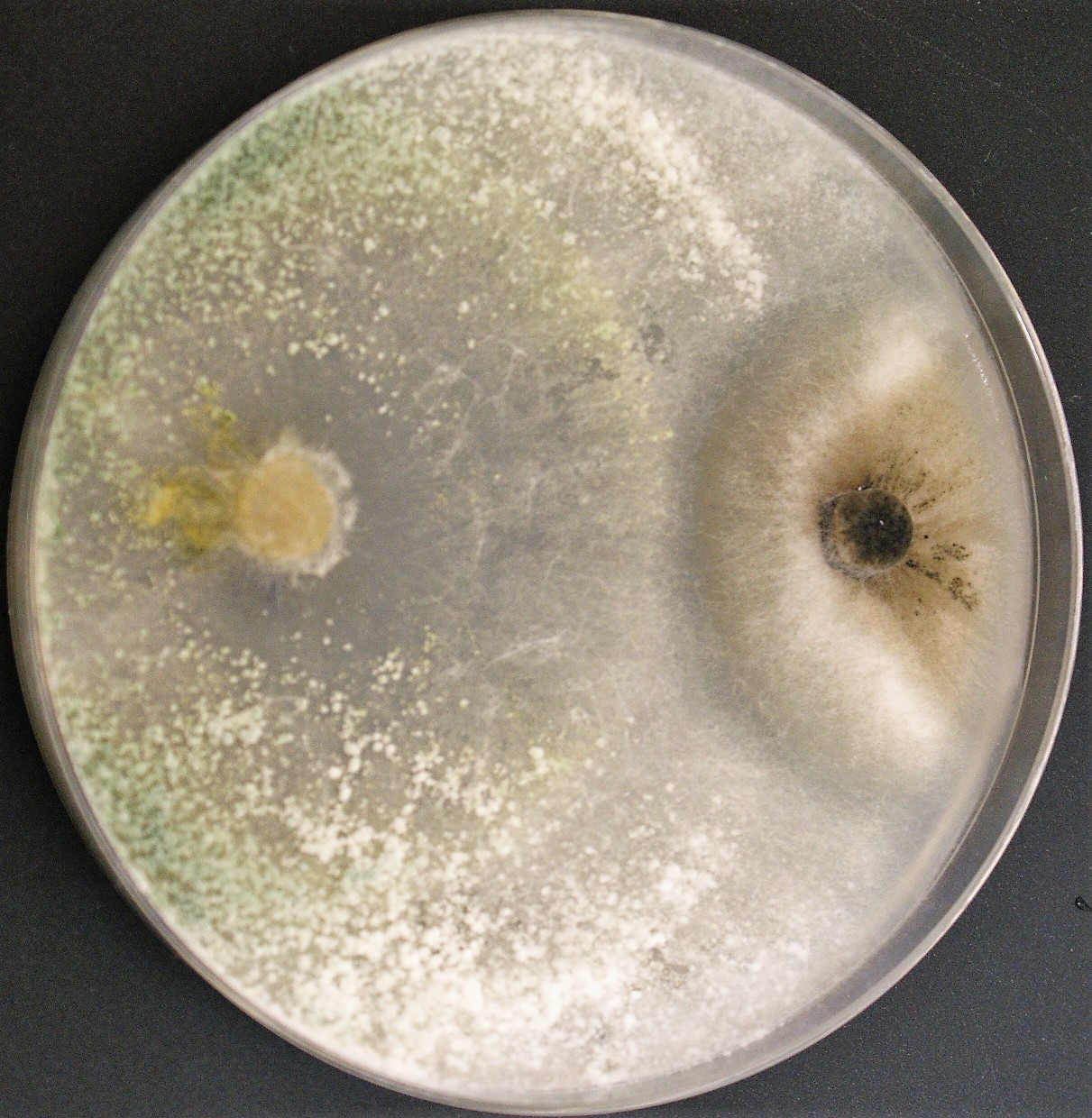

Supplement: Supplemental Information 1 — Images depict comparative growth of multiple fungal variants (labelled), thereby indicating the inhibitory potential of Trichoderma. [file peerj-11-15392-s001.zip › Photos/T.virideI-Colletotrichum.jpg]

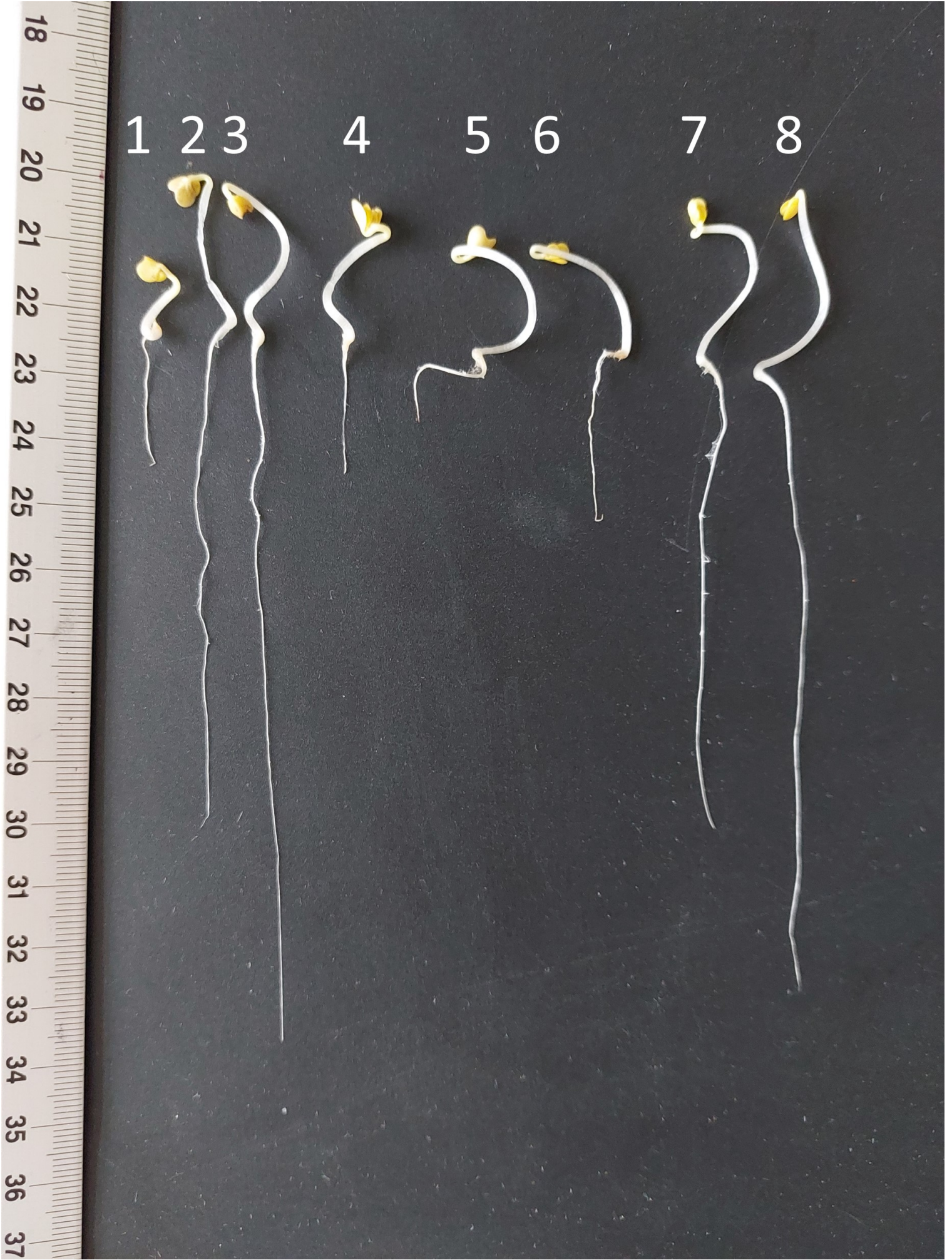

Supplement: Supplemental Information 2 — 1 - uncoated seeds, 2 - methylcellulose-chitin-Trichoderma viride I, 3 - methylcellulose-chitin-T. viride II, 4 - methylcellulose, 5 - chitin, 6 - Methylcellulose–chitin, 7 - T. viride I, and 8 - T. viride II. [file peerj-11-15392-s002.pdf]
